# The Clinical Features and Predictive Factors of Nocturnal Enuresis in Adult Women

**DOI:** 10.3389/fmed.2021.744214

**Published:** 2022-01-17

**Authors:** Qi-Xiang Song, Jiayi Li, Yiyuan Gu, Lei Xu, Paul Abrams, Wei Xue

**Affiliations:** ^1^Department of Urology, Renji Hospital, Shanghai Jiao Tong University School of Medicine, Shanghai, China; ^2^Department of Urology, Changhai Hospital, Naval Medical University, Shanghai, China; ^3^Bristol Urological Institute, Southmead Hospital, Bristol, United Kingdom

**Keywords:** nocturnal enuresis, lower urinary symptoms, nocturia, nocturnal polyuria, bladder diary, urodynamics, detrusor overactivity, detrusor underactivity

## Abstract

**Objective:**

Our current knowledge on nocturnal enuresis (NE) in adults is scarce due to its uncommon nature. The present study was designed to investigate symptom characteristics and risk factors of NE in adult women to improve the current clinical understanding and management of this rare disease.

**Methods:**

Over a 3-year period, we enrolled 70 adult women who complained of bedwetting, with a frequency of at least once per week and a symptom duration of 3 months or longer. Patients were excluded if they had known pregnancy, current urinary tract infection, untreated malignancies, anatomical abnormalities, and irregular sleep cycle. The International Consultation on Incontinence Modular Questionnaire—female lower urinary tract symptoms and bladder diary were employed to appraise lower urinary tract symptoms and voiding behavior. Urodynamics was performed to assess the bladder function. A linear regression analysis was applied to determine potential risk factors for NE frequency.

**Results:**

Among the recruited subjects, comorbidities and lower urinary tract symptoms were frequently reported. On bladder diary, patients commonly presented with nocturnal polyuria (NP), reduced nocturnal voided volumes (RNVVs), or both. Urodynamics revealed multiple dysfunctions, namely, detrusor overactivity (DO), urodynamic stress incontinence (USI), reduced compliance, bladder outlet obstruction, detrusor underactivity (DU), and simultaneous DO and DU. Patients with more frequent NE (≥4/week) demonstrated markedly increased body mass index, more comorbid conditions, worse incontinence symptoms, NP or NP plus RNVVs, reduced compliance, and poorer voiding possibly owing to DU. Whereas, RNVVs alone and worse overactive bladder-related parameters were associated with milder NE. Multivariate analysis indicated that frequency/urgency quality of life, incontinence symptom, NP + RNVVs, poor flow, increased bladder sensation, USI, and simultaneous detrusor overactivity plus DU were independent risk factors for NE severity.

**Conclusion:**

NE in adult women may have both urological and non-urological pathophysiology. Imbalanced circadian urine production, jeopardized continence mechanisms, overactive bladder, and DU-induced poor voiding are major factors that contribute to the pathogenesis of NE in adult women. Focused treatments on restoring these functions should be individually considered.

## Introduction

As a common pediatric condition, nocturnal enuresis (NE) in children or adolescents is usually self-limited, with an estimated prevalence of 15% at 5 years dropping to 1–2% after 15 years (1). When it comes to adults, the overall incidence of NE is 0.02–2.3% according to multiple reports with slightly different diagnostic criteria (2–4). Despite being uncommon, NE should not be neglected, as it has a considerable negative impact on an individual's quality of life (QoL), self-esteem, mental health, and family relationships (5). However, our current knowledge is scarce, due to, ① few publications with small sample sizes; ② inconsistent definitions and criteria for patients with NE; and ③ lack of assessments of voiding pattern and urine output by bladder diary.

Recently, we did a comprehensive evaluation of NE in adult men, suggesting that obesity and deteriorated bladder emptying capability due to neurogenic deficiency or bladder outlet obstruction (BOO), as well as nocturnal polyuria (NP) with reduced nocturnal voided volumes (RNVVs) were independent risk factors for NE severity (6). However, women are prone to have a group of different lower urinary tract symptoms (LUTS) and systemic conditions, which may be related to NE giving a gender-specific pathophysiology and etiology. For example, the incidence of stress urinary incontinence (SUI) is much more prevalent in women, due to relatively shorter urethra and weaker bladder outlet resistance compare with that in men. Therefore, in this study, we attempted to investigate the features of female patients with NE, to promote our current clinical understanding and management of this infrequent disease.

## Materials and Methods

### Study Design

The current study is a parallel branch of our previous cross-sectional analysis of NE in adult men (6). Starting from September 2017, adult women (18 years and older) complaining of bedwetting during night-time sleep were investigated. Upon recruitment, past medical history and the presence of LUTS were documented. A validated International Consultation on Incontinence Modular Questionnaire—female lower urinary tract symptoms (ICIQ-FLUTS) (long form) and a 3-day International Consultation on Incontinence Modular Questionnaire—bladder diary (ICIQ-BD) were used to quantitatively evaluate storage and voiding symptoms (7, 8). The frequency of NE was recorded for seven consecutive nights. Urodynamics was performed to objectively assess the lower urinary tract function. With the approval from the institutional review board and after verbal consent from each subject, investigations were performed by a single urologist using an agreed protocol.

### Patients

The diagnostic criteria for NE were in accordance with the International Continence Society (ICS) report and our previous publication (6, 9). In brief, bedwetting occurs during the main sleep period, with a frequency of at least once per week and a symptom duration of 3 months or longer. This allows us to focus on patients with more bothersome symptoms, instead of sporadic NE which is difficult to capture. We excluded patients with known pregnancy, current urinary tract infection, untreated pelvic/urinary tract malignancies, and anatomical abnormalities (ectopic kidney, duplication of ureter, urinary fistula, etc.), which may cause unintended urine leakage. Subjects with serious medical conditions, such as acute heart and kidney failure and recent cerebrovascular accident, were considered ineligible to participate. In addition, night shift workers or those with irregular sleep cycles were excluded, so that the definition of “night-time” can be consistently applied to all subjects.

### Assessments

The ICIQ-FLUTS was categorized into three domains: the frequency/urgency domain scores from 0 to 11 for degree and from 0 to 30 for QoL; the voiding domain scores from 0 to 12 for degree and from 0 to 30 for QoL; and the incontinence/leakage domain scores from 0 to 20 for degree, from 0 to 50 for QoL, and from 0 to 19 for general leakage impact. Patients were required to complete the forms in the office with professionals' assistance to fully understand each question, if necessary.

A 3-day bladder diary was obtained from each patient to calculate the mean voided volume, maximum voided volume, 24-h frequency, nocturnal frequency, 24-h urine volume, and nocturnal urine volume based on the ICS definitions (9, 10). To differentiate the causes of nocturia, nocturnal polyuria index (NPi), and nocturnal bladder capacity index (NBCi) were calculated as previously described, that is, an NPi ≥20% for age <35 years or NPi ≥33% for age >35 years is used to define NP, while an NBCi >1.3 is indicative of RNVVs (also known as reduced nocturnal bladder capacity) (11). In addition, global polyuria was defined as 24-h urine production exceeding 40 ml/kg.

All subjects underwent uroflowmetry twice with a minimal voided volume of 150 ml or more, followed by a transabdominal ultrasound measurement of post-void residuals (PVR). Urodynamic studies were carried out using a water-filled pressure conduction system (Triton, Laborie Medical Technology, Canada) in compliance with the ICS Good Urodynamic Practice Guidelines (12). With both a 7Fr double-lumen transurethral catheter and a transrectal balloon catheter in place, the bladder was filled by room temperature saline at a medium rate (30–50 ml/min) with the patient in a seating position. Increased bladder sensation was defined as the presence of a first sensation <150 ml. Reduced bladder compliance was defined as Δ bladder pressure/Δ bladder volume <40 ml/cm H_2_O. The Solomon-Greenwell nomogram was used, and potential BOO was defined as a female bladder outlet obstruction index (P_det_Q_max_ – 2.2^*^Q_max_) above 5 (13). The female-modified bladder contractility index was calculated as P_det_Q_max_ + Q_max_, with a value <30 as an indication of detrusor underactivity (DU) (14). Additionally, the simultaneous presence of detrusor overactivity (DO) during storage and DU during the voiding phase was identified, formerly known as detrusor hyperactivity with impaired contractility (15).

### Statistical Analysis

Data were analyzed by a specialist blinded to the medical records of subjects using the SPSS software (version 25, IBM, NY, USA). Quantitative variables were compared using either independent *t*-tests or Mann–Whitney *U*-tests based on their normality. The statistical significance between nominal variables was detected using Pearson's chi-squared tests or Fisher's exact tests. A panel of indices was included in the univariate linear regression analysis, with *p* ≤ 0.3 as filter threshold for entering the subsequent multivariable analysis model, in which the probability value to enter level was set at 0.05 and removal level was 0.1.

## Results

In this study, 81 eligible consecutive female patients with NE were identified from September 2017 to March 2021. Among them, eight were excluded due to either lack of urodynamics data or absent/incomplete bladder diary records. One with uncontrolled cervical cancer and two with poor health conditions were excluded. In total, complete data from 70 patients were enrolled for analysis.

### General Characteristics of Subjects

The recruited subjects covered a wide range of age distribution from 18 to 80 years, with 40% having a body mass index (BMI) ≥24 kg/m^2^, and 72.9% reported systemic comorbidities (Table 1). Less than 50% were being treated with oral medications, such as α-blocker, antimuscarinics, β3 agonist, and Chinese herb medicine, either alone or in combination. For most of them, the reason for taking medications was to relieve daytime overactive bladder symptoms and/or voiding problems, rather than treating bedwetting. The etiology of NE can be stratified into either primary (55.7%) or secondary (44.3%) onset, based on their past history. The former was further divided into primary persistent (8.6%) and primary recurrent (47.1%) according to their symptom continuity since childhood, while the latter could be classified as neurogenic (Parkinson's disease, Alzheimer's disease, encephalitis, spinal cord injury, and meningomyelocele), drug-related (anti-depressant, hypnotics, and sedatives), and surgery-related (hysterectomy, colorectal surgery, and pelvic floor reconstruction) causes of NE (5, 16).

**Table 1:** The demographic data of adult women with NE.

	**Cases (*N* = 70)**	**Percentage (%)**
Age (years), (mean ± SD, range)	56.8 ± 13.7	18–80
<50	18	25.7
≥50, <70	38	54.3
≥70	14	20.0
BMI (kg/m^2^), (mean ± SD, range)	23.3 ± 4.2	16.2–33.8
<18.5	9	12.9
≥18.5, <24	33	47.1
≥24	28	40.0
**Comorbidities**		
Hypertension	32	45.7
Diabetes mellitus	18	25.7
Hyperlipemia	17	24.3
Coronary heart disease	12	17.1
None	19	27.1
One comorbidity	28	40.0
Two comorbidities	14	20.0
Three comorbidities or more	9	12.9
**Oral medications**		
None	38	54.3
Alpha-blocker	14	20.0
Antimuscarinics	9	12.9
Beta-3 agonist	4	5.7
Chinese herb medicine	7	10.0
Monotheraphy	28	40.0
Combination therapy	4	5.7
**Other treatments**		
Acupuncture	4	5.7
Intermittent self-catheterization	4	5.7
**Enuresis classification**		
Primary persistent	6	8.6
Primary recurrent	33	47.1
Secondary (neurogenic)	10	14.3
Secondary (drug-related)	5	7.1
Secondary (surgery-related)	16	22.9

*BMI, body mass index; NE, nocturnal enuresis*.

### Overall LUTS, Bladder Diary, and Urodynamics

**Figure 1:**
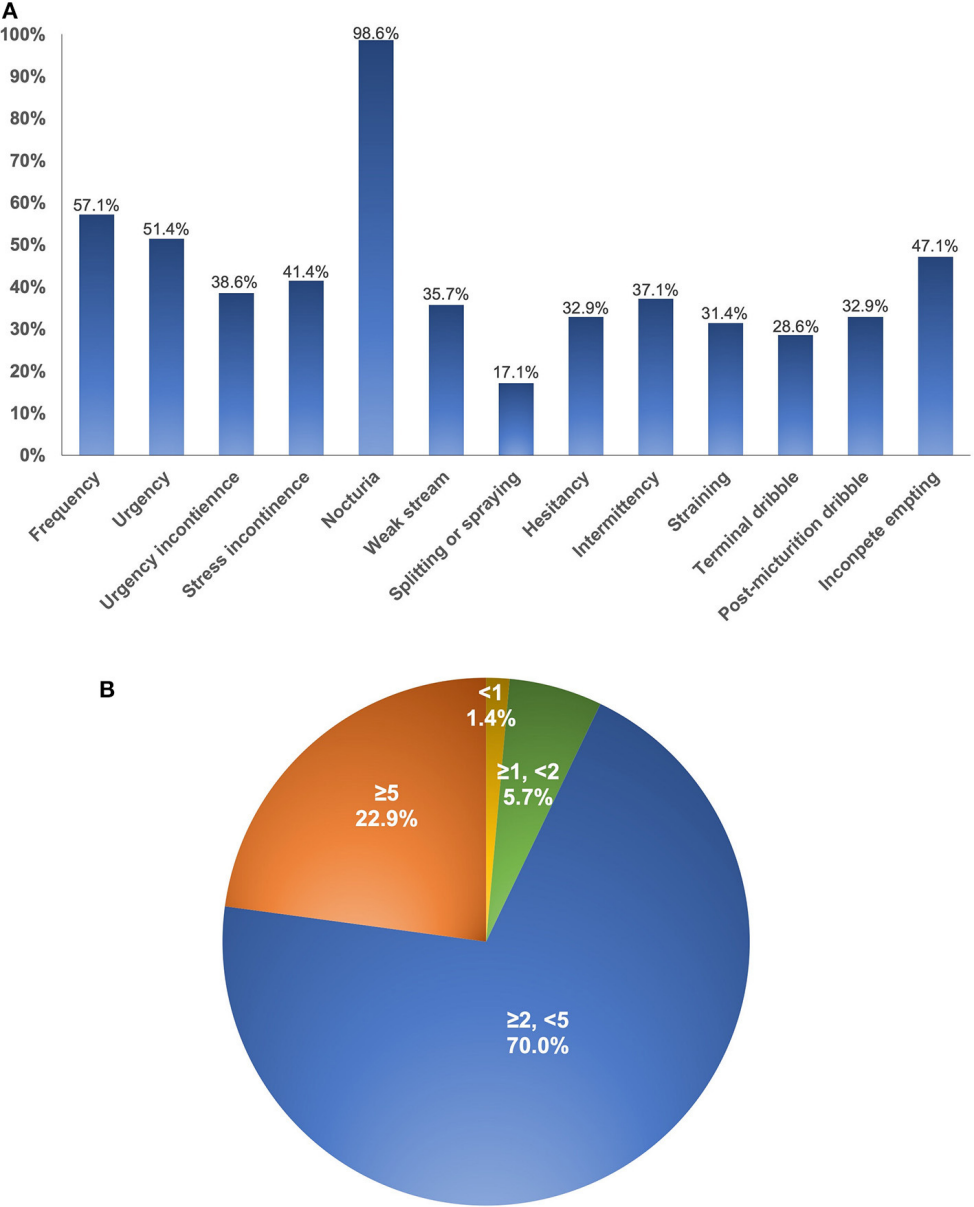
The incidence of each lower urinary tract symptom subtype **(A)** and the distribution of nocturnal episodes **(B)**. Nocturia is defined as wake up at least 1 time to void during night-time sleep.

Nocturia (98.6%), frequency (57.1%), and urgency (51.4%) were the top three prevalent LUTS in this group of patients (Figure 1). Among them, as high as 72.9% suffered at least two wake-up voids per night. In addition, there was a relatively high incidence of overall daytime incontinence, such as stress (41.4%), urgency (38.6%), and mixed (15.7%) subtypes. In addition, painful voiding (10.0%), vulvodynia/vaginodynia (8.6%), and bladder region pain (7.1%) were reported by a small number of patients.

The ICIQ-FLUTS suggested quite high scores in the incontinence and urine leakage impact domains, reflecting the serious symptoms that bother both daytime incontinence and NE (Table 2). The calculations derived from ICIQ-BD demonstrated that the incidence of NP, RNVVs, and both were 44.3, 70.0, and 31.4%, respectively. The uroflowmetry suggested that 12.9% had a Q_max_ <15 ml/s, and 17.1% had a PVR of at least 100 ml. On urodynamics, during the filling phase, 68.6% had increased bladder sensation, 40.0% had DO, 28.6% demonstrated urodynamic stress incontinence (USI), and 24.3% had reduced compliance; during the voiding phase, the potential BOO, DU, and co-existing DO/DU were identified in 21.4, 20.0, and 17.1% of subjects, respectively.

**Table 2:** International Consultation on Incontinence Modular Questionnaire—female lower urinary tract symptoms (ICIQ-FLUTS) scores, bladder diary, and urodynamic parameters.

	**Mean ±SD/median/*n***	**Range/IQR/%**
**ICIQ-FLUTS**		
Frequency and urgency^#^	6.4 ± 4.7	2.0–11.0
Frequency and urgency QoL^#^	16.7 ± 8.1	0.0–40.0
Voiding^∧^	6.5	1.0–10.0
Voiding QoL^∧^	15.5	2.3–25.3
Incontinence^∧^	9.5	5.0–15.0
Incontinence QoL^∧^	24.0	14.5–38.5
General leakage impact^∧^	11.0	7.0–16.0
**Bladder diary**		
Mean voided volume (ml)^∧^	201.9	148.9–226.0
24-h maximum voided volume (ml)^#^	330.5 ± 84.3	120.0–550.0
24-h frequency (voids)^∧^	11.2	9.6–13.2
Nocturnal frequency (voids)^∧^	3.0	3.0–4.3
24-h urine volume (ml)^#^	2155.1 ± 480.7	1,139.0–3,483.6
Nocturnal urine volume (ml)^∧^	580.6	470.2–749.4
24-h polyuria^†^	20	28.6%
NP^†^	31	44.3%
RNVVs^†^	49	70.0%
NP only^†^	9	12.9%
RVVs only^†^	27	38.6%
NP + RNVVs^†^	22	31.4%
NE episodes in a week^∧^	3.0	2.0–5.0
**Uroflowmetry**		
Q_max_ (ml/s)^∧^	22.9	17.9–33.6
<10^†^	3	4.3%
≥15^†^	61	87.1%
Voided volume (ml)^∧^	363.1	226.9–472.3
PVR (ml)^∧^	50.0	8.8–80.0
≥100^†^	12	17.1%
**Urodynamics**		
First sensation (ml)^∧^	121.9	75.2–158.7
<150^†^	48	68.6%
≥250^†^	3	4.3%
Cystometric capacity (ml)^#^	325.8 ± 143.7	65.2–769.1
Compliance (ml/cmH_2_O)^∧^	70.4	39.8–132.6
<40^†^	17	24.3%
DO^†^	28	40.0%
Single DO^†^	14	20.0%
Terminal DO^†^	3	4.3%
Phasic DO^†^	11	15.7%
P_det_Q_max_ (cmH_2_O)^∧^	32.4	21.6–41.3
Q_max_ (ml/s)^∧^	17.3	13.7–22.9
BOOIf^∧^	−7.3	−17.6–0.5
>5^†^	15	21.4%
BCI^∧^	50.5	37.6–63.8
≤ 30^†^	14	20.0%
DO + DU^†^	12	17.1%
USI^†^	20	28.6%

*Total frequency and urgency sub-score ranges from 0 to 11; frequency and urgency quality of life (QoL) sub-score ranges from 0 to 30; voiding sub-score ranges from 0 to 12; voiding QoL sub-score ranges from 0 to 30; incontinence sub-score ranges from 0 to 20; incontinence QoL sub-score ranges from 0 to 50; general leakage impact sub-score ranges from 0 to 19*;

# *ormally distributed continuous variables are presented as mean ± SD and range*.

∧ *Non-normally distributed continuous variables are presented as median and interquartile range (IQR)*.

† *Categorical variables are presented as number and rate. BCI, bladder contractility index; BOOIf, bladder outlet obstruction index for female; DO, detrusor overactivity; DU, detrusor underactivity; ICIQ-FLUTS, International Consultation on Incontinence Modular Questionnaire—female lower urinary tract symptoms; IQR, interquartile range; NE, nocturnal enuresis; NP, nocturnal polyuria; PVR, post-void residuals; QoL, quality of life; RNVVs, reduced nocturnal voided volume; SD, standard deviation; USI, urodynamic stress incontinence*.

### Subgroup Analysis

When stratified according to the NE episodes (Table 3), we identified that subjects with more frequent NE (≥4/week) were associated with a significantly higher BMI, more comorbidities, higher incontinence/urine leakage scores, the presence of NP and NP + RNVVs, reduced compliance, and poorer bladder emptying possibly owing to DU. On the contrary, patients with milder symptoms (<4/week) predominantly manifested RNVVs only and much worse overactive bladder-related parameters (higher frequency/urgency QoL score, increased bladder sensation, and reduced cystometric capacity). Echoing the above findings, further subgroup analysis indicated that patients with higher BMI or NP had significantly more comorbidities, higher incontinence/urine leakage scores, larger nocturnal urine production, and poorer voiding than their corresponding comparison groups (Supplementary Table). Not surprisingly, the presence of RNVVs was accompanied by dramatically higher frequency/urgency scores, lower mean voided volume, and increased global and nocturnal frequency. Further comparisons between patients with secondary and primary NE revealed that the former not only had significantly more bedwetting episodes, higher BMI, more comorbidities, and worse incontinence symptoms, but also demonstrated significantly poorer compliance, weaker detrusor contractility, and higher residual volume on urodynamics.

**Table 3:** Subgroup analysis stratified by enuresis episodes.

	**NE <4/week** **(*n* = 36)**	**NE≥4/week** **(*n* = 34)**	***P***
Age (years)	56.5 (16.3)	56.9 (11.2)	0.89^#^
BMI (km/m^2^)	20.7 (2.7)	26.0 (3.7)	<0.01^#^*
**Comorbid conditions**			
None	19 (52.8)	0 (0.0)	<0.01^†^*
One	16 (44.4)	12 (35.3)	
Two	1 (2.8)	13 (38.2)	
Three or more	0 (0.0)	9 (26.5)	
**ICIQ-FLUTS**			
Frequency and urgency	6.0 (3.0)	6.0 (3.0)	0.70^∧^
Frequency and urgency QoL	20.4 (8.2)	12.7 (5.9)	<0.01^#^*
Voiding	4.0 (8.0)	8.0 (10.0)	0.07^∧^
Voiding QoL	14.0 (20.0)	20.0 (27.0)	0.37^∧^
Incontinence	5.0 (5.0)	15.0 (9.0)	<0.01^∧^*
Incontinence QoL	15.0 (10.0)	39.0 (18.0)	<0.01^∧^*
General leakage impact	7.5 (5.0)	16.0 (6.0)	<0.01^∧^*
**Bladder diary**			
Mean voided volume (ml)	185.0 (71.2)	207.3 (87.7)	0.38^∧^
Max voided volume (ml)	317.5 (80.4)	344.2 (87.3)	0.19^#^
24-h frequency (voids)	10.9 (3.9)	11.5 (3.3)	0.29^∧^
Nocturnal frequency (voids)	3.0 (2.0)	4.0 (2.0)	0.09^∧^
Nocturnal urine volume (ml)	492.0 (165.4)	735.3 (251.7)	<0.01^#^*
NP	7 (19.4)	24 (70.6)	<0.01^†^*
RNVVs	26 (72.2)	23 (67.6)	0.68^†^
NP only	3 (8.3)	6 (17.6)	0.30^†^
RNVVs only	22 (61.1)	5 (14.7)	<0.01^†^*
NP + RNVVs	4 (11.1)	18 (52.9)	<0.01^†^*
NE episodes in a week	2.0 (2.0)	5.0 (3.0)	<0.01^∧^*
**Uroflowmetry**			
Q_max_ (ml/s)	29.2 (17.1)	20.6 (7.8)	<0.01^∧^*
Voided volume (ml)	400.1 (251.7)	336.1 (246.1)	0.76^∧^
PVR (ml)	17.5 (50.0)	80.0 (114.0)	<0.01^∧^*
**Urodynamics**			
First sensation (ml)	80.1 (79.5)	145.5 (67.2)	<0.01^∧^*
Cystometric capacity (ml)	282.1 (128.4)	372.1 (146.3)	<0.01^#^*
Compliance (ml/cmH_2_O)	80.6 (118.4)	46.8 (106.6)	0.048^∧^*
BOOIf	−10.1 (16.2)	−2.8 (21.0)	0.09^∧^
BCI	58.7 (26.7)	42.3 (27.0)	<0.01^#^*

# *Normally distributed continuous variables are presented as mean (SD) and are compared using Student's t-test*.

∧ *Non-normally distributed continuous variables are presented as median (IQR) and are compared using Mann–Whitney U-test*.

† *Categorical variables are presented as number (rate) and are compared using the chi-squared test or Fisher's exact test. Asterisks indicate statistically significant differences between the two subgroups. BCI, bladder contractility index; BMI, body mass index; BOOIf, bladder outlet obstruction index for female; ICIQ-FLUTS, International Consultation on Incontinence Modular Questionnaire—female lower urinary tract symptoms; NE, nocturnal enuresis; NP, nocturnal polyuria; PVR, post-void residuals; QoL, quality of life; RNVVs, reduced nocturnal voided volume*.

### Identification of Risk Factors

In general, 34 potentially related variables were brought into univariate linear regression analysis, which revealed a number of risk factors, which can be categorized into the following groups: ① systematic factors: BMI, comorbid conditions, and surgery-related secondary NE; ② lower urinary tract symptoms: frequency and urgency QoL, voiding symptom, and incontinence/urine leakage scores on ICIQ-FLUTS; ③ bladder diary indices: nocturnal frequency; nocturnal urine volume; the presence of NP and NP + RNVVs; ④ uroflowmetry/urodynamic parameters: Q_max_, PVR, cystometric capacity, increased sensation, USI, DU, concomitant DO with DU, and reduced compliance (Table 4). Therewith, based on the 28 eligible parameters, multivariable analysis was performed, suggesting high incontinence/urine leakage scores; the presence of NP + RNVVs; poor flow; USI; and co-existing DO/DU, were independent risk factors for a more severe NE. In contrast, those with serious complaints of frequency and urgency and increased bladder sensation suffered from less bothersome NE.

**Table 4:** Potential risk factors associated with nocturnal enuresis severity.

	**Univariate linear regression**			**Multivariate linear regression**		
	**B**	**β**	***P*-value**	**B**	**β**	***P*-value**
Age	−0.01	−0.05	0.69	-	-	-
BMI	0.35	0.68	<0.01	-	-	0.67
The presence of comorbid conditions	2.40	0.50	<0.01	-	-	0.71
The numbers of comorbid conditions	1.50	0.73	<0.01	-	-	0.44
**Potential causes of secondary NE**						
Neurogenic-related	1.21	0.20	0.09	-	-	0.70
Drug-related	−1.29	−0.15	0.20	-	-	0.64
Surgery-related	2.37	0.46	<0.01	-	-	0.20
**ICIQ-FLUTS**						
Frequency and urgency	−0.04	−0.04	0.77	-	-	-
Frequency and urgency QoL	−0.11	−0.40	<0.01	−0.05	−0.18	<0.01*
Voiding	0.13	0.26	0.03	-	-	0.51
Voiding QoL	0.02	0.13	0.30	-	-	0.61
Incontinence	0.32	0.86	<0.01	0.13	0.35	<0.01*
Incontinence QoL	0.13	0.86	<0.01	-	-	0.75
General leakage impact	0.33	0.79	<0.01	0.08	0.19	0.046*
**Bladder diary**						
Mean voided volume	<0.01	−0.02	0.87	-	-	-
Maximum voided volume	<0.01	0.10	0.41	-	-	-
24-h frequency	0.16	0.23	0.06	-	-	0.69
Nocturnal frequency	0.39	0.32	<0.01	-	-	0.85
24-h urine volume	<0.01	0.19	0.12	-	-	0.59
Nocturnal urine volume	0.01	0.61	<0.01	-	-	0.35
The presence of 24-h polyuria	0.35	0.07	0.55	-	-	-
The presence of NP	2.27	0.52	<0.01	-	-	0.88
The presence of RNVVs	0.74	0.16	0.20	-	-	0.33
The presence of NP + RNVVs	2.61	0.56	<0.01	0.79	0.17	<0.01*
**Uroflowmetry**						
Q_max_	−0.10	−0.52	<0.01	−0.03	−0.16	<0.01*
PVR	0.01	0.63	<0.01	-	-	0.17
**Urodynamics**						
Cystometric capacity	0.01	0.33	<0.01	-	-	0.98
The presence of increased sensation	−1.82	−0.39	<0.01	−0.72	−0.15	<0.01*
The presence of detrusor overactivity	0.87	0.20	0.10	-	-	0.29
The presence of USI	2.52	0.53	<0.01	0.65	0.14	0.02*
The presence of potential BOO	0.42	0.08	0.51	-	-	-
The presence of DU	2.57	0.48	<0.01	-	-	0.10
The presence of DO + DU	3.06	0.54	<0.01	1.52	0.27	0.04*
The presence of reduced compliance	1.90	0.38	<0.01	-	-	0.88

*Twenty-eight parameters (p ≤ 0.3 in univariate analysis) were included in the multivariable analysis. The Durbrin-Watson test value was 1.355, R = 0.935, R^2^ = 0.875, and the adjusted R^2^ = 0.856. Asterisks indicate statistical significance. BMI, body mass index; BOO, bladder outlet obstruction; DO, detrusor overactivity; DU, detrusor underactivity; ICIQ-FLUTS, International Consultation on Incontinence Modular Questionnaire—female lower urinary tract symptoms; NP, nocturnal polyuria; QoL, quality of life; RNVVs, reduced nocturnal voided volume; USI, urodynamic stress incontinence*.

## Discussion

Nocturnal enuresis in adults has long been overlooked and inadequately investigated due to its uncommon nature. Patients usually choose to endure and may be unwilling to turn to healthcare professionals even until bedwetting becomes bothering, possibly due to embarrassment and lack of awareness. Unlike NE in children, which may be owing to delayed maturation of arousal mechanisms or unestablished diurnal rhythm of vasopressin secretion, NE in adults is often associated with systemic diseases and LUTS, or secondary to surgeries and certain drugs, making the diagnosis and management more challenging (17). Our previous investigation in adult men with NE has identified a number of clinically significant risk factors using parameters derived from questionnaires, bladder diary, and urodynamics (6). In this parallel study, comparable approaches were used to analyze the features and risk factors of NE in adult women.

There is a relatively high percentage of women with increased BMI (≥24 kg/m^2^), which is associated with more frequent NE, consistently with our findings from that in men and reports from Song et al. (6) and Madhu et al. (18). As to the causes of secondary NE, we found that 11 out of 70 women had previous hysterectomy, although surgical onset only serves as a risk factor for NE episodes in the univariate analysis. According to the previous reports, NE was not an uncommon symptom after radical hysterectomy, usually coupled with day-time incontinence (19, 20). During cervical dissection and lymphadenectomy, damage to the hypogastric plexus, splanchnic nerves, and paraurethral supporting structures could give rise to quite a high incidence of post-surgical LUTS, which can be ameliorated using a nerve-sparing technique (21, 22).

The overall incidence of daytime incontinence was 64.3% in women with NE, with 41.4% suffering from SUI, which was unsurprisingly higher than that in men due to gender difference (6). Importantly, we further corroborated that incontinence-related subjective symptom scores on ICIQ-FLUTS and USI are independent predictors for NE frequency (18). One can deduce that the already reduced urethral resistance in patients with SUI may get worse during sleep, causing more disastrous bedwetting. Therefore, it seems possible that treating the underlining SUI in women could potentially alleviate or eradicate NE, but further research on this issue would be useful (23).

The clinical use of bladder diary has seldomly been reported in adult patients with NE. In our last publication, the ICIQ-BD has been verified as a valuable tool in assessing the pathophysiology of NE, providing unique insights into diurnal and nocturnal voiding patterns, in particular the identification of RNVVs and NP (6). Consistent findings in women suggested that more patients suffered worse NE if they had NP, whereas the presence of RNVVs only, was associated with fewer NE episodes. The evidence of NP + RNVVs, as an independent predictor for NE degree, suggested an indisputable role for excessive night-time urine volume in the pathogenesis of NE, justifying the treatment modalities that target restoration of homeostatic nocturnal voided volumes and urine production for adult patients with NE.

In this study, urodynamic abnormalities during the storage and voiding phase were frequently reported by adult NE women (18, 24–26). In addition to the evidence that patients with NE had significantly more DO and mixed incontinence during urodynamics, we found that overactive bladder symptom-related QoL and increased bladder sensation are negative risk factors for NE severity (18). Moreover, we revealed that poor flow, high PVR, and low detrusor contractility were related to a more frequent NE, but only higher Q_max_ negatively predict NE episodes. These findings are distinct from the investigation in men, which suggested increased PVR, possibly due to BOO strongly predicted NE frequency (6). Notably, co-existing DO/DU, as a seemingly paradoxical condition, serves as an independent predictor for NE severity. It is worth mentioning that DO with DU can be generated as a result of neurogenic deficiencies, affective disorders, gastrointestinal dysfunctions, and metabolic syndrome, etc., sustaining the multifactorial nature of both co-existing DO/DU and NE (27).

There are a few limitations to consider, starting with the limited number of patients, even with over 3 years of scrutinized screening. Despite the low prevalence of adult NE, we adopted strict criteria, recruiting patients with significant and persistent bedwetting, so that the analysis can focus on those with more bothering symptoms, and therefore, likely to request clinical intervention. Furthermore, incontinence was only assessed with questionnaires and verified during urodynamics, without quantitative data from pad tests or leak point pressure. In addition, the status of NE during the adolescents and childhood life of patients was unavailable, making the analysis impossible to investigate its connection with adult-onset NE.

In conclusion, adding to our previous knowledge on adult men with NE, this parallel study provides particular insights into female subjects, suggesting excessive nocturnal urine production, jeopardized continence mechanisms, the presence of overactive bladder, and impaired voiding function due to DU are the major factors contributing to the pathogenesis of NE in adult women. Therefore, personalized treatments for female patients with NE should be considered, centering on either ameliorating SUI, promoting bladder storage function, improving detrusor contractility, or restoring the homeostasis of circadian urine production.

## Data Availability Statement

The raw data supporting the conclusions of this article will be made available by the authors, without undue reservation.

## Ethics Statement

This study involving the participation of patients was reviewed and approved by the Institutional Review Board of Renji Hospital. The patients/participants provided their verbal consent to take part in this study.

## Author Contributions

Q-XS, JL, PA, and WX designed the study. Q-XS and JL acquired, interpreted, and analyzed the data. LX helped with data collection and performed the urodynamic studies. YG was responsible for database administration and conducting statistical analysis. Q-XS and JL drafted the manuscript, which was critically revised by PA and WX. All authors have contributed substantially to the intellectual content of this paper.

## Funding

This work was supported by Renji Hospital Natural Science Foundation Promoting Project, RJTJ22-MS-015.

## Conflict of Interest

PA was a consultant for Recordati, Astellas, and Ipsen and a lecturer for Astellas, Pfizer, and Sun Pharma. The remaining authors declare that the research was conducted in the absence of any commercial or financial relationships that could be construed as a potential conflict of interest.

## Publisher's Note

All claims expressed in this article are solely those of the authors and do not necessarily represent those of their affiliated organizations, or those of the publisher, the editors and the reviewers. Any product that may be evaluated in this article, or claim that may be made by its manufacturer, is not guaranteed or endorsed by the publisher.

## Supplementary Material

Supplementary Table Additional subgroup analysis. ^#^Normally distributed continuous variables are presented as mean (SD) and are compared using Student's *t*-test. ^∧^Non-normally distributed continuous variables are presented as median (interquartile range [IQR]) and are compared using Mann–Whitney *U*-test. ^†^Categorical variables are presented as number (rate) and are compared using the chi-squared test or Fisher's exact test. Asterisks indicate statistically significant differences between the two subgroups. BCI, bladder contractility index; BMI, body mass index; BOOIf, bladder outlet obstruction index for female; ICIQ-FLUTS, International Consultation on Incontinence Modular Questionnaire—female lower urinary tract symptoms; NE, nocturnal enuresis; NP, nocturnal polyuria; PVR, post-void residuals; QoL, quality of life; RNVVs, reduced nocturnal voided volume.

## References

[1] DiBianco JM Morley C Al-Omar O. Nocturnal enuresis: a topic review and institution experience. Avicenna J Med. (2014) 4:77–86. 10.4103/2231-0770.140641 25506580 PMC4251068

[2] Hirasing RA van Leerdam FJ Bolk-Bennink L Janknegt RA. Enuresis nocturna in adults. Scand J Urol Nephrol. (1997) 31:533–6. 10.3109/00365599709030657 9458510

[3] Yeung CK Sihoe JD Sit FK Bower W Sreedhar B Lau J. Characteristics of primary nocturnal enuresis in adults: an epidemiological study. BJU Int. (2004) 93:341–5. 10.1111/j.1464-410X.2003.04612.x 14764133

[4] Sakamoto K Blaivas JG. Adult onset nocturnal enuresis. J Urol. (2001) 165:1914–7. 10.1016/S0022-5347(05)66241-6 11371880

[5] Katz EG MacLachlan LS. Nocturnal enuresis in the adult. Curr Urol Rep. (2020) 21:31. 10.1007/s11934-020-00983-2 32506170

[6] Song QX Wang L Cheng X Hao Y Liu Z Abrams P. The clinical features and predictive factors of nocturnal enuresis in adult men. BJU Int. (2020) 126:472–80. 10.1111/bju.15126 32475016 PMC7589435

[7] Bright E Cotterill N Drake M Abrams P. Developing and validating the international consultation on incontinence questionnaire bladder diary. Eur Urol. (2014) 66:294–300. 10.1016/j.eururo.2014.02.057 24647230

[8] Huang L Zhang SW Wu SL Ma L Deng XH. The Chinese version of ICIQ: a useful tool in clinical practice and research on urinary incontinence. Neurourol Urodyn. (2008) 27:522–4. 10.1002/nau.20546 18351586

[9] Hashim H Blanker MH Drake MJ Djurhuus JC Meijlink J Morris V. International Continence Society (ICS) report on the terminology for nocturia and nocturnal lower urinary tract function. Neurourol Urodyn. (2019) 38:499–508. 10.1002/nau.23917 30644584

[10] Hashim H Drake MJ. Basic concepts in nocturia, based on international continence society standards in nocturnal lower urinary tract function. Neurourol Urodyn. (2018) 37:S20–4. 10.1002/nau.23781 30070389

[11] Weiss JP. Nocturia: “do the math”. J Urol. (2006) 175:S16–8. 10.1016/S0022-5347(05)00312-5 16458734

[12] Rosier P Schaefer W Lose G Goldman HB Guralnick M Eustice S. International continence society good urodynamic practices and terms 2016: urodynamics, uroflowmetry, cystometry, and pressure-flow study. Neurourol Urodyn. (2017) 36:1243–60. 10.1002/nau.23124 27917521

[13] Solomon E Yasmin H Duffy M Rashid T Akinluyi E Greenwell TJ. Developing and validating a new nomogram for diagnosing bladder outlet obstruction in women. Neurourol Urodyn. (2018) 37:368–78. 10.1002/nau.23307 28666055

[14] Griffiths D. Detrusor contractility–order out of chaos. Scand J Urol Nephrol Suppl. (2004) 215:93–100. 10.1080/03008880410015426 15545203

[15] Resnick NM Yalla SV. Detrusor hyperactivity with impaired contractile function. An unrecognized but common cause of incontinence in elderly patients. JAMA. (1987) 257:3076–81. 10.1001/jama.257.22.3076 3586227

[16] Akhavizadegan H Locke JA Stothers L Kavanagh A. A comprehensive review of adult enuresis. Can Urol Assoc J. (2018) 13:282–7. 10.5489/cuaj.5485 30273117 PMC6737735

[17] Kuwertz-Broking E von Gontard A. Clinical management of nocturnal enuresis. Pediatr Nephrol. (2018) 33:1145–54. 10.1007/s00467-017-3778-1 28828529

[18] Madhu CK Hashim H Enki D Drake MJ. Risk factors and functional abnormalities associated with adult onset secondary nocturnal enuresis in women. Neurourol Urodyn. (2017) 36:188–91. 10.1002/nau.22912 26473752

[19] Kadar N Saliba N Nelson JH. The frequency, causes and prevention of severe urinary dysfunction after radical hysterectomy. Br J Obstet Gynaecol. (1983) 90:858–63. 10.1111/j.1471-0528.1983.tb09328.x 6615743

[20] Rayome RG King C King T. Patient with nocturnal enuresis and stress incontinence after previous hysterectomy and radiation therapy for cervical cancer. J Wound Ostomy Continence Nurs. (1995) 22:64–7. 10.1097/00152192-199501000-00015 7704146

[21] Kietpeerakool C Aue-Aungkul A Galaal K Ngamjarus C Lumbiganon P. Nerve-sparing radical hysterectomy compared to standard radical hysterectomy for women with early stage cervical cancer (stage Ia2 to IIa). Cochrane Database Syst Rev. (2019) 2:CD012828. 10.1002/14651858.CD012828.pub2 30746689 PMC6370917

[22] Rob L Halaska M Robova H. Nerve-sparing and individually tailored surgery for cervical cancer. Lancet Oncol. (2010) 11:292–301. 10.1016/S1470-2045(09)70191-3 20202614

[23] Campbell P Li W Money-Taylor J Davies J Gray T Radley S. Nocturnal enuresis: prevalence and associated LUTS in adult women attending a urogynaecology clinic. Int Urogynecol J. (2017) 28:315–20. 10.1007/s00192-016-3099-0 27480535

[24] Lee D Dillon BE Lemack GE. Adult onset nocturnal enuresis: identifying causes, cofactors and impact on quality of life. Low Urin Tract Symptoms. (2018) 10:292–6. 10.1111/luts.12183 28675645

[25] Yeung CK Sihoe JD Sit FK Diao M Yew SY. Urodynamic findings in adults with primary nocturnal enuresis. J Urol. (2004) 171:2595–8. 10.1097/01.ju.0000112790.72612.0a 15118427

[26] Wadie BS. Primary nocturnal enuresis persistent to adulthood, functional evaluation. Neurourol Urodyn. (2004) 23:54–7. 10.1002/nau.10161 14694458

[27] Mancini V Tarcan T Serati M Wyndaele M Carrieri G Abrams P. Is coexistent overactive-underactive bladder (with or without detrusor overactivity and underactivity) a real clinical syndrome? ICI-RS 2019. Neurourol Urodyn. (2020) 39(Suppl. 3):S50–9. 10.1002/nau.24311 32032454

