# Supplementary material for: The Clinical Features and Predictive Factors of Nocturnal Enuresis in Adult Women
Source: Front Med (Lausanne). 2022 Jan 17;8:744214. doi: 10.3389/fmed.2021.744214 (PMC8801585; doi:10.3389/fmed.2021.744214)
Supplement: Supplementary Table — Additional subgroup analysis. #Normally distributed continuous variables are presented as mean (SD) and are compared using Student's t-test. ∧Non-normally distributed continuous variables are presented as median (interquartile range [IQR]) and are compared using Mann–Whitney U-test. †Categorical variables are presented as number (rate) and are compared using the chi-squared test or Fisher's exact test. Asterisks indicate statistically significant differences between the two subgroups. BCI, bladder contractility index; BMI, body mass index; BOOIf, bladder outlet obstruction index for female; ICIQ-FLUTS, International Consultation on Incontinence Modular Questionnaire—female lower urinary tract symptoms; NE, nocturnal enuresis; NP, nocturnal polyuria; PVR, post-void residuals; QoL, quality of life; RNVVs, reduced nocturnal voided volume. [file Table_1.DOCX]

**Supplementary table: additional subgroup analysis**

|  | **Age** | | | **BMI** | | | **NP** | | | **RNVVs** | | | **NE classification** | | |
| --- | --- | --- | --- | --- | --- | --- | --- | --- | --- | --- | --- | --- | --- | --- | --- |
|  | **<65**  **(n=45)** | **≥65**  **(n=25)** | **P** | **<24**  **(n=42)** | **≥24**  **(n=28)** | **P** | **No**  **(n=39)** | **Yes**  **(n=31)** | **P** | **No**  **(n=21)** | **Yes**  **(n=49)** | **P** | **Primary**  **(n=39)** | **Secondary**  **(n=31)** | **P** |
| **Age (years)** | 50.0 (14.0) | 70.0 (9.0) | **<0.01**^^^ * | 56.1 (14.8) | 57.8 (12.2) | 0.62^#^ | 58.7 (13.3) | 54.1 (14.5) | 0.17^#^ | 54.0 (16.4) | 57.9 (12.8) | 0.29^#^ | 56.3 (15.8) | 57.2 (11.5) | 0.81^#^ |
| **BMI (km/m^2^)** | 23.2 (4.4) | 23.4 (3.8) | 0.88^#^ | 20.9 (3.7) | 26.8 (5.0) | **<0.01**^^^ * | 21.2 (5.5) | 24.4 (6.4) | **<0.01**^^^ * | 23.4 (3.4) | 23.2 (4.5) | 0.91^#^ | 22.1 (3.0) | 24.8 (4.9) | **0.01^#^ *** |
| **Comorbid conditions** |  |  |  |  |  |  |  |  |  |  |  |  |  |  |  |
| None | 14 (31.1) | 5 (20.0) | 0.35^†^ | 17 (40.5) | 2 (7.1) | **<0.01**^†^ * | 17 (43.6) | 2 (6.4) | **<0.01**^†^ * | 6 (28.6) | 13 (26.5) | 0.98^†^ | 16 (41.0) | 3 (9.7) | **<0.01**^†^ * |
| One | 17 (37.8) | 11 (44.0) |  | 19 (45.2) | 9 (32.1) |  | 13 (33.3) | 15 (48.4) |  | 10 (47.6) | 18 (36.7) |  | 16 (41.0) | 12 (38.7) |  |
| Two | 10 (22.2) | 4 (16.0) |  | 4 (9.5) | 10 (35.7) |  | 7 (17.9) | 7 (22.6) |  | 4 (19.0) | 10 (20.4) |  | 7 (17.9) | 7 (22.6) |  |
| Three or more | 4 (8.9) | 5 (20.) |  | 2 (4.8) | 6 (25.1) |  | 2 (5.1) | 7 (22.6) |  | 1 (4.8) | 8 (16.3) |  | 0 (0.0) | 9 (29.1) |  |
| **ICIQ-FLUTS** |  |  |  |  |  |  |  |  |  |  |  |  |  |  |  |
| Frequency & urgency | 6.2 (2.0) | 6.8 (2.5) | 0.31^#^ | 6.0 (3.0) | 6.0 (4.0) | 0.36^ | 6.0 (4.0) | 6.0 (3.0) | 0.10^^^ | 5.5 (2.3) | 6.9 (2.0) | **0.01**^#^ * | 6.6 (2.3) | 6.3 (2.0) | 0.53^#^ |
| Frequency & urgency QoL | 15.9 (5.7) | 18.1 (11.2) | 0.36^#^ | 17.9 (8.9) | 14.8 (6.5) | 0.10^#^ | 17.9 (8.5) | 15.1 (7.4) | 0.14^#^ | 12.0 (7.0) | 18.0 (12.0) | **0.02**^^^ * | 18.8 (8.5) | 13.9 (6.7) | **0.01^#^ *** |
| Voiding | 8.0 (8.0) | 6.0 (9.0) | 0.30^^^ | 5.5 (7.0) | 8.0 (11.0) | 0.25^^^ | 4.0 (9.0) | 8.0 (10.0) | 0.10^^^ | 5.0 (11.0) | 7.0 (9.0) | 0.66^^^ | 5.0 (9.0) | 7.0 (8.0) | 0.39^^^ |
| Voiding QoL | 16.0 (19.0) | 13.0 (28.0) | 0.62^^^ | 15.0 (18.0) | 17.0 (30.0) | 0.77^^^ | 15.0 (26.0) | 18.0 (22.0) | 0.73^^^ | 15.0 (30.0) | 16.0 (19.0) | 0.92^^^ | 15.0 (26.0) | 17.0 (19.0) | 0.91^^^ |
| Incontinence | 10.0 (10.0) | 8.0 (9.0) | 0.66^^^ | 6.0 (6.0) | 14.0 (9.0) | **<0.01**^^^ * | 6.0 (6.0) | 13.0 (9 .0) | **<0.01**^^^ * | 8.0 (7.0) | 10.0 (11.0) | 0.46^^^ | 6.0 (6.0) | 15.0 (11.0) | **<0.01**^^^ * |
| Incontinence QoL | 26.0 (28.0) | 22.0 (18.0) | 0.53^ | 17.5 (16.0) | 37.5 (20.0) | **<0.01**^^^ * | 17.0 (16.0) | 37.0 (21.0) | **<0.01**^^^ * | 20.0 (18.0) | 26.0 (26.0) | 0.18^ | 20.0 (14.0) | 37.0 (28.0) | **<0.01**^^^ * |
| General leakage impact | 12.0 (9.0) | 11.0 (9.0) | 0.90^^^ | 8.0 (6.0) | 15.5 (5.0) | **<0.01**^^^ * | 8.0 (8.0) | 16.0 (8.0) | **<0.01**^^^ * | 10.0 (9.0) | 12.0 (9.0) | 0.34^^^ | 10.0 (8.0) | 16.0 (11.0) | **<0.01**^^^ * |
| **Bladder diary** |  |  |  |  |  |  |  |  |  |  |  |  |  |  |  |
| Mean voided volume (ml) | 197.4 (46.3) | 175.4 (52.0) | 0.07^#^ | 184.5 (52.9) | 197.0 (43.0) | 0.30^#^ | 205.3 (75.0) | 201.4 (89.2) | 0.16^^^ | 211.1 (6646) | 185.3 (86.0) | **0.04**^^^ * | 199.3 (49.3) | 177.2 (47.0) | 0.06^#^ |
| Max voided volume (ml) | 336.1 (79.5) | 320.4 (93.1) | 0.46^#^ | 321.2 (90.0) | 344.4 (74.2) | 0.26^#^ | 330.3 (84.5) | 330.8 (85.3) | 0.98^#^ | 345.7 (81.3) | 323.9 (85.5) | 0.33^#^ | 331.3 (74.9) | 329.4 (96.0) | 0.93^#^ |
| 24-hour frequency (voids) | 11.1 (3.5) | 11.4 (4.3) | 0.12^^^ | 11.3 (4.5) | 11.2 (2.8) | 0.99^^^ | 11.2 (3.9) | 11.3 (2.8) | 0.42^^^ | 9.8 (3.3) | 11.8 (3.4) | **<0.01**^^^ * | 10.4 (3.7) | 11.8 (3.1) | **0.01**^^^ * |
| Nocturnal frequency (voids) | 3.0 (1.5) | 3.0 (2.0) | 0.60^^^ | 3.0 (3.0) | 3.5 (1.0) | 0.53^^^ | 3.0 (2.0) | 4.0 (2.0) | **0.03**^^^ * | 2.0 (1.5) | 4.0 (2.0) | **<0.01**^^^ * | 3.0 (2.0) | 4.0 (2.0) | 0.11^^^ |
| Nocturnal urine volume (ml) | 593.0 (340.5) | 550.9 (259.8) | 0.30^^^ | 537.1 (203.2) | 696.9 (166.2) | **<0.01**^#^ * | 510.1 (192.2) | 715.4 (156.6) | **<0.01**^#^ * | 553.4 (238.0) | 621.4 (186.4) | 0.20^#^ | 544.0 (187.0) | 672.7 (204.4) | **<0.01**^#^ * |
| NP | 21 (46.7) | 10 (40.0) | 0.59^†^ | 13 (31.0) | 18 (64.3) | **<0.01**^†^ * | 0 (0.0) | 31 (100.0) | **<0.01**^†^ * | 9 (42.9) | 22 (44.9) | 0.82^†^ | 11 (28.2) | 20 (64.5) | **<0.01**^†^ * |
| RNVVs | 30 (66.7) | 19 (76.0) | 0.41^†^ | 30 (71.4) | 19 (67.9) | 0.75^†^ | 27 (69.2) | 22 (71.0) | 0.88^†^ | 0 (0.0) | 49 (100.0) | **<0.01**^†^ * | 25 (64.1) | 24 (77.4) | 0.23 |
| NP only | 9 (20.0) | 0 (0.0) | **0.02**^†^ * | 5 (11.9) | 4 (14.3) | 0.78^†^ | 0 (0.0) | 9 (29.0) | **<0.01**^†^ * | 9 (42.9) | 0 (0.0) | **<0.01**^†^ * | 4 (10.3) | 5 (16.4) | 0.50 |
| RNVVs only | 18 (40.0) | 9 (36.0) | 0.74^†^ | 22 (52.4) | 5 (17.9) | **<0.01**^†^ * | 27 (69.2) | 0 (0.0) | **<0.01**^†^ * | 0 (0.0) | 27 (55.1) | **<0.01**^†^ * | 18 (46.2) | 9 (29.0) | 0.14 |
| NP + RNVVs | 12 (26.7) | 10 (40.0) | 0.25^†^ | 8 (19.0) | 14 (50.0) | **<0.01**^†^ * | 0 (0.0) | 22 (71.0) | **<0.01^†^ *** | 0 (0.0) | 22 (44.9) | **<0.01**^†^ * | 7 (17.9) | 15 (48.4) | **<0.01^†^ *** |
| **NE episodes in a week** | 4.0 (4.0) | 3.0 (3.0) | 0.39^^^ | 2.5 (2.0) | 5.0 (3.0) | **<0.01**^^^ * | 3.0 (3.0) | 5.0 (3.0) | **<0.01^^^ *** | 4.0 (3.0) | 3.0 (4.0) | 0.33^^^ | 3.0 (2.0) | 5.0 (4.0) | **<0.01^^^ *** |
| **Uroflowmetry** |  |  |  |  |  |  |  |  |  |  |  |  |  |  |  |
| Q_max_ (ml/s) | 23.0 (19.0) | 22.8 (14.6) | 0.57^^^ | 28.7 (20.2) | 17.8 (10.0) | **<0.01**^^^ * | 23.9 (20.1) | 19.3 (11.2) | **0.02**^^^ * | 23.0 (19.3) | 22.5 (13.2) | 0.55^^^ | 25.1 (14.7) | 19.3 (21.0) | 0.10^^^ |
| Voided volume (ml) | 341.5 (296.8) | 377.5 (158.1) | 0.71^^^ | 370.3 (222.3) | 344.2 (341.6) | 0.89^ | 425.3 (232.4) | 302.7 (178.4) | **0.03**^^^ * | 431.6 (295.6) | 346.9 (216.2) | 0.27^^^ | 377.5 (254.1) | 341.5 (217.1) | 0.32^^^ |
| PVR (ml) | 50.0 (63.0) | 20.0 (80.0) | 0.08^^^ | 30.0 (53.0) | 80.0 (100.0) | **<0.01**^^^ * | 40.0 (70.0) | 75.0 (120.0) | **<0.01**^^^ * | 30.0 (70.0) | 50.0 (68.0) | 0.12^^^ | 40.0 (70.0) | 70.0 (140.0) | **<0.01**^^^ * |
| **Urodynamics** |  |  |  |  |  |  |  |  |  |  |  |  |  |  |  |
| First sensation (ml) | 115.9 (87.9) | 128.4 (101.0) | 0.94^^^ | 89.6 (79.7) | 154.1 (72.1) | **<0.01**^^^ * | 109.1 (93.9) | 131.2 (86.9) | 0.32^^^ | 123.7 (88.8) | 115.9 (90.4) | 0.85^^^ | 120.0 (90.7) | 123.7 (90.5) | 0.37^^^ |
| Cystometric capacity (ml) | 337.6 (152.6) | 304.5 (126.4) | 0.36^#^ | 303.5 (148.3) | 359.2 (132.1) | 0.11^#^ | 307.0 (126.9) | 349.5 (161.4) | 0.22^#^ | 317.8 (118.3) | 329.2 (154.3) | 0.76^#^ | 305.0 (283.6) | 352.0 (151.6) | 0.18^#^ |
| Compliance (ml/cmH_2_O) | 68.8 (83.0) | 77.3 (154.9) | 0.20^^^ | 75.4 (74.5) | 61.2 (160.6) | 0.51^^^ | 83.5 (88.1) | 49.6 (106.6) | **0.03**^^^ * | 83.5 (112.7) | 66.5 (91.5) | 0.13^^^ | 83.0 (134.5) | 49.6 (105.5) | **0.046^^^ *** |
| BOOIf | -4.5 (20.3) | -8.8 (13.0) | 0.42^^^ | -11.2 (17.6) | -2.8 (14.9) | 0.08^^^ | -7.3 (20.0) | -7.3 (17.9) | 0.79^^^ | -3.6 (22.8) | -7.3 (16.7) | 0.88^^^ | -6.1 (17.2) | -7.7 (23.1) | 0.62^^^ |
| BCI | 51.6 (31.1) | 49.4 (14.9) | 0.79^^^ | 58.5 (25.1) | 41.3 (23.1) | **<0.01**^^^ * | 55.5 (24.0) | 42.6 (31.1) | **0.03**^^^ * | 53.7 (21.3) | 47.1 (29.6) | 0.13^^^ | 53.7 (20.1) | 41.9 (31.1) | **0.03**^^^ * |
